# 112 What’s your emergency? Overview of mental health and sleep disorders among emergency medical dispatchers in a French 112 call center

**DOI:** 10.1186/s13049-024-01228-8

**Published:** 2024-06-10

**Authors:** Louise Giaume, Y. Daniel, A. Jimenez, G. Burlaton, D. Jost, M. Petitclerc, F. Briche, P. Hertgen, P. Amzstut, P. Mercier, C. Louyot, M. Trousselard, S. Travers

**Affiliations:** 1 French Military Biomedical Research, Brétigny-sur-Orge, France; 2 Emergency Medical Department, Paris Fire Brigade, 1 Place Jules Renard, Paris, 75017 France; 3 Val-de-Grâce Military Health Academy, 1 Place Alphonse Laveran, Paris, 75005 France

**Keywords:** Emergency medical dispatchers, Occupational health, Sleep disorders, Stress, Post-traumatic stress

## Abstract

**Background:**

Emergency medical dispatchers (EMD) experience significant occupational stress. Medical dispatching includes call-taking, triage, dispatch, and providing callers gesture guidance to the victims. Every decision has a major impact on the patient’s outcome. Chronic exposure to stress and potentially traumatic situations, combined with night shifts can impact the stress response and physical health of staff.

**Objectives:**

To evaluate the prevalence of mental health and sleep disorders among EMD personnel working in a 112-call center, prior to an evidence-based prevention intervention (primary outcome); and to assess the relationship between health outcomes and DM (secondary outcome).

**Methods:**

We conducted a descriptive, monocentric study with 109 EMD. HAD Anxiety (HAD-A) and Depression (HAD-D) scores, and the PTSD checklist for DSM-5 (PCL-5) were used to explore mental health disorders. The Epworth Sleepiness Scale, and other analog scales were used to explore sleep disorders. DM resources were assessed using the Freiburg Mindfulness Inventory (FMI), and its Presence and Acceptance subscales.

**Results:**

A total of 72% of the EMD working in the call center were included. Of these, 16.6% had moderate anxiety disorder, and 6.4% had an anxiety disorder (Mean HAD-A: 6.05 ± 2.88). Furthermore, 16.6% had a moderate depression disorder, and 6.4% had a depression disorder (Mean HAD-D: 4.28 ± 3.28), and 16% had symptoms of PTSD (Mean PCL-5: 17.57 ± 13.67). Turning to sleep, 39% may suffer from excessive daytime sleepiness (EDS), and 10% had confirmed EDS (Mean Epworth score 10.47 ± 4.41). Finally, 39% had moderate insomnia, and 59% had severe insomnia (Mean insomnia: 13.84 ± 5.77.). Medium-strength negative correlations were found between mental health and DM (FMI scores and sub-scores: −0.48 < *r* < − 0.29; 0.001 < *p* < 0.004); and a positive correlation was found between DM and daytime awareness (0.22 < *r* < 0.26; 0.01 < *p* < 0.03).

**Conclusion:**

The prevalence of depression, symptoms of PTSD, and sleep disorders in our sample of EMD is significant, and confirms findings reported in the literature. The EMD population may benefit from specific, multi-level interventions that target mindfulness, sleep, and ergonomics to improve their mental and physical health.

## Background

Emergency medical dispatchers (EMD) experience significant stress in the workplace, and several teams have reported greater prevalence of chronic disease [1]. Medical dispatching is a highly complex procedure that includes call-taking, triage (categorizing emergency calls), the dispatch of appropriate resources, and the provision of guidance and instructions to callers. Every decision that is taken has a major impact on the patient’s outcome, as emergency call conditions are often time-critical [2]. Every day, EMD staff are exposed to stressful and potentially traumatic situations such as death, life-threatening incidents, and psychological, physical, and social distress, sometimes involving young children [3]. They must not only listen to and reassure the caller, but also assess the situation, and provide a rapid and appropriate response—without seeing the victim [4]. In some situations, a language barrier or extreme distress means that EMD are unable to instruct the caller in what to do to help the victim. In the long term, responding to calls, the mental load, and repeated exposure to stressful and potentially traumatic situations, combined with physiological disruption linked to working night shifts has a significant impact on EMD’s stress response and their physical health [5].

Chronic exposure to stress can lead to operational fatigue, burnout, anxiety and depressive disorder, and PTSD [6]. In the professional context, exposure can take the form of electronic media, such as emergency call-taking. Repeated exposure to critical incidents could have a cumulative effect, which increases the risk for PTSD [7]. In addition to this stressful professional context, EMD’s physical health is impacted by sleep disorders and chronic fatigue (due to working night shifts), physical injuries such as musculoskeletal disorders (due to outdated equipment and poor ergonomics), high noise levels, and poor lighting, which are correlated with higher cortisol levels [8]. All of these factors contribute to high turnover among EMDs, who often choose to move to other less stressful professions [9].

112 is the European emergency number that will get you straight through to the emergency services. You can dial free of charge from fixed and mobile phones everywhere in the EU. In France, the capital’s emergency 112 call center is staffed by military firefighters from the Paris Fire Brigade (PFB), the fire department of the City of Paris. All year round, 24 h a day, the PFB deploys 24 emergency dispatchers who handle 2350 calls each day (i.e. over 100 calls per dispatcher per day) from callers within the City of Paris, and three adjacent departments. These personnel are former active firefighters who have experience of providing fire protection services and basic life support to the population. There are three roles: (i) *dispatch*, (ii) *incoming calls*, and (iii) *re-assessment*. Working hours are divided into shifts. During the COVID-19 pandemic, they were faced with additional stress, and were asked to adopt new procedures.

While several factors have been found to positively or negatively affect work-related outcomes for EMD, an understudied element in this professional context is dispositional mindfulness (DM), also known as mindfulness trait. DM is characterized by an awareness that emerges by purposefully paying nonjudgmental attention, in the present moment, to an unfolding experience [10]. It has been conceptualized as a trait understood as the ability to be mindful in everyday life, regardless of events, stably across time, and is associated with various positive physical and psychological health factors, such as efficient emotional and stress regulation [11]. Mindfulness techniques are recognized as a useful way to facilitate the development of DM and other abilities that help people deal with their daily stressors, and alleviate the consequences for health and well-being [12].

In 2021, an evidence-based prevention intervention called FIRECARE was launched. Based on mindfulness, cardiac coherence, and positive psychology, this intervention proved to be useful in preventing burnout among the PFB’s prehospital caregivers [13]. This outcome encouraged us to extend the intervention to the PFB’s EMD. However, before proceeding, we decided to evaluate the prevalence of mental health disorders (anxiety, depression, PTSD, and sleep disorders) among EMDs working in the Paris 112 call center after COVID (primary outcome). The secondary outcome was to assess the relationship between health outcomes and DM, based on the hypothesis that greater DM is associated with better health.

## Methods

### Study design

This is a descriptive and monocentric study. In November and December 2022, a link to an anonymous questionnaire hosted by the online application Wepi Pro was emailed to the professional account of all operators working in the 112-call center staffed by the PFB. Three email campaigns were run.

### Data acquisition

A socio-biographical questionnaire was used to collect standard sociodemographic in the PFB.

The 14-item Hospital Anxiety and Depression scale (HAD) is used to screen for anxiety and depressive disorders. A score ≤ 7 indicates no symptoms; 8–11 suggests doubtful symptomatology; and ≥ 11 indicates clear symptomatology [14].

The PTSD Checklist for the DSM-5 (PCL-5) is a 20-item self-administered questionnaire. Subjects rate their symptoms on a scale ranging from 0 (*not at all*) to 4 (*extremely*) with respect to the preceding month. PCL-5 scores range from 0 to 80 [15].

The 8-item Epworth Sleepiness Scale (ESS) aims to self-assess the risk of daytime sleepiness in daily life. A score of 0–10 is normal, 11–16 indicates potential excessive daytime sleepiness (EDS), and > 16 is evidence of EDS [16].

An 11-item self-assessment questionnaire was used to assess the quality of sleep. The first 4 answers are given using a visual scale, and the last 7 use a scale ranging from *none* to *extreme* [17].

The Freiburg Mindfulness Inventory (FMI) is a 14-item self-report mindfulness questionnaire. It measures the person’s ability to live fully in the present moment and assesses their level of self-perception with respect to two subfactors: Presence and Acceptance. The mean score for the French version is 38.98 ± 5.43 [18].

Resilience was assessed with the Connor-Davidson Resilience scale (CD-RISC) is a 25-item self-report questionnaire that assesses several aspects of resilience. This instrument uses a 5-point Likert-type response scale ranging from 0 (*Not at all true*) to 4 (*True most of the time*) [19].

### Data analysis

Descriptive statistics were expressed as mean ± SD, or as a percentage. Pearson’s correlations were run to explore the relationship between health outcomes and DM. For all analyses, significance was set at *p* < 0.05.

## Results

The questionnaire was sent to 150 EMD working for the PFB. Of these, 109 responded (72%) and were included in the study. Sociodemographic data are presented in Table 1.

**Table 1:** Sociodemographic data for the total sample. Results are presented as mean *±* standard deviation or as a percentage. 72% of the PFB’ EMD respond to the questionnaires

Sociodemographic data	
*Total of EMD in the PFB n= 150*	*n* = 109
Sex: male	98% (*n* = 107)
Age (mean ± standard deviation)	31.6 ± 5.4
Years working for the Paris Fire Brigade	10.8 ± 5.3
Years working at the 112 call center	3.1 ±2.2
Experience of a stressful event	59%
Resilience scale (M ± SD)	93.51 ± 11.46
CD-RISC score ≤ 80	6%

### Mental health data are shown in table 2

**Table 2:** Mental health data for the total sample. Results are presented as a percentage, or as mean ± standard deviation. 72% of the PFB’ EMD respond to the questionnaires

Mental health data	
*Total of EMD in the PFB n= 150*	*n* = 109
HAD Anxiety (M ± SD) - No anxiety: score < 8 - Moderate anxiety disorder: score 8–10 - Anxiety disorder: score ≥ 11	6.05 ± 2.88 77% 16.6% 6.4%
HAD Depression (M ± SD) - No depression: score < 8 - Moderate depression disorder: score 8–10 - Depression disorder: score ≥ 11	4.28 ± 3.28 79% 17.4% 3.6%
Post traumatic stress disorder (CD-PCL 5) (M ± SD) PCL-5 >33	17.57 ± 13.67 16%

The mean (SD) HAD Anxiety score was 6.05 (2.88). Hence, 77% of the sample had no anxiety disorder, 16.6% had a moderate anxiety disorder, and 6.4% had an anxiety disorder. The mean (SD) HAD Depression score was 4.28 (3.28). Here, 79% had no depression disorder, 16.6% had a moderate depression disorder, and 6.4% had a depression disorder. The mean (SD) PTSD score was 17.57 (13.67). Thus, 16% of participants reported PTSD symptoms. Medium to strong positive correlations were found between HAD Anxiety, HAD Depression, and PCL-5 scores (0.34 < *r* < 0.63; *p* < 0.001).

### Sleep disorder data are shown in table 3

**Table 3:** Sleep disorder data for the total sample. Results are presented as a percentage, or as mean ± standard deviation. 72% of the PFB’ EMD respond to the questionnaires

Sleep disorder data	
*Total of EMD in the PFB n= 150*	*n* = 109
Epworth Sleepiness Scale (M ± SD) Normal (score 0–10) Potential EDS (> 10) Confirmed EDS (> 16)	10.47 ±4.41 51% 39% 10%
Intensity of sleep disorder (M ± SD)	
Visual scale (0–10) (M ± SD) Visual scale > 5	4.49 ± 2.89 53%
Quality of sleep (M ± SD)	
Visual scale (0–10) (M ± SD) Visual scale ≤ 5	4.27 ± 2.80 56%
Poor awareness during the day (M ± SD)	
Visual scale (0–10) (M ± SD) Visual scale ≤ 5	6.25 ± 2.35 26%
Insomnia (M ± SD) No insomnia: ISI < 8 Moderate insomnia: 8 ≤ ISI < 15 Severe insomnia: ISI ≥ 15	13.84 ± 5.77 12% 39% 49%

The mean (SD) score on the ESS was 10.47 (4.41). Hence, 51% of the sample experience no daytime sleepiness, 39% might have EDS, and 10% have confirmed EDS.

The mean (SD) of the analog *intensity of sleep disorder scale* was 4.49 (2.89). This indicates that 53% of the sample scored above 5. The mean (SD) of the analog *poor quality of sleep* scale was 4.27 (2.8). Hence, 56% of the sample scored 5 or below. The mean (SD) of the analog *poor awareness during the day* scale was 6.25 (2.35). Here, 26% of the sample scored 5 or below.

The mean (SD) insomnia score was 13.84 (5.77). This suggests that 12% of the sample did not suffer from insomnia, 39% had moderate insomnia, and 49% had severe insomnia.

Medium to strong correlations were found between insomnia, intensity of sleep disorder, and quality of sleep (*p* < 0.001). Higher scores for insomnia were consistent with higher intensity sleep disorder, and poorer-quality sleep. Poorer awareness during the day was associated with poorer-quality sleep, higher intensity sleep disorder, and a high level of insomnia (*p* < 0.001). Concerning the Epworth Sleepiness Scale, higher scores were associated with poorer sleep quality (*p* = 0.033), lower awareness during the day (*p* = 0.005), and a higher level of insomnia (*p* = 0.01). Furthermore, almost all sleep variables were significantly correlated with health outcomes. Sleep disorder intensity, and poor awareness during the day were both correlated with the PCL-5 score (SD intensity *p* = 0.001; Awareness *p* < 0.001). Sleep quality and insomnia were both correlated respectively with anxiety, depression, and PTSD (*sleep quality: p* = 0.001, *p* = 0.008, *p* = 0.002; *insomnia p* = 0.025, *p* = 0.001, *p* < 0.001).

The mean (SD) overall FMI score was 37.07 (6.37). The mean (SD) score for Awareness was 18.48 (3.67), and 18.78 (3.45) for Presence.

Medium-strength, negative correlations were found between HAD Anxiety, HAD Depression, and PCL-5 scores, and mindfulness (sub)scores (− 0.48 < *r* < − 0.29; 0.001 < *p* < 0.004). Weak positive correlations were found between awareness during the day, and mindfulness (sub)scores (0.22 < *r* < 0.26; 0.01 < *p* < 0.03).

## Discussion

The present study shows a significant prevalence of anxiety (moderate: 16.6%, acute: 6.4%), depression (moderate: 17.4%, acute: 3.6%), and PTSD (16%) among the sampled EMD. We Strong correlations were found between psychopathological data and sleep disorder data.

Our results are similar to those reported in the literature. For example, a multicentric French study report that 15% of medical dispatch assistants in an emergency medical care center (i.e., SAMU-center) suffered from full PTSD (PCL-5 ≥ 34) [20]. The latter group were more likely to have adopted stress reduction strategies (alcohol, drugs, medication) (13% vs. 2%), had more food disorders (80.5% vs. 38%), more sleeping disorders (75.5% vs. 21%), more anxiety (67% vs. 17%), and took more sick leave (13% vs. 4%) than the remainder of the sample. Similarly, a study from across the United States reported PTSD prevalence of 18–24%, and major depression of 23.9% among a sample of 911 telecommunicators [6]. Finally, a study of a German EMD cohort reported prevalences of PTSD, anxiety, and depressive disorder as, respectively, 11.3% (PC-PTSD-5), 7%, and 15.5% [1]. Although the prevalence of anxiety and depression among EMD seems to be similar to the rate in the general French population [21], the prevalence of PTSD is high [22]. On the other hand, resilience seems to be particularly high among our sample, compared to the general population [23]. While resilience has many definitions and features, it is broadly understood as the capacity to cope with stressful and potentially traumatic events. It is also seen as a psychobiological factor that determines the individual’s response to adverse life events [24]. Higher psychological resilience is often correlated with better mental health, and can protect against PTSD [25].

A significant rate of sleep disorders was observed in our cohort: 39% of the sample may have EDS, and 10% have confirmed EDS. Furthermore, 56% complained of poor sleep quality, 39% have moderate insomnia, and 49% have severe insomnia. In this context, it is relevant to consider shift work disorder (SWD). SWD is a circadian rhythm disorder associated with the shift schedule, and involves insomnia and/or excessive sleepiness [26]. It has been an established disorder for over 20 years and is listed in the *International Classification of Sleep Disorders*. According to the literature, up to 50% of paramedics experience SWD, and 19.4% were found to be at high risk for SWD after 6 months of working in emergency services [27]. Pre-shift work depression levels have been found to be a significant risk factor for 6-month SWD [27]. SWD is also associated with workplace injuries, motor vehicle accidents, increased sick leave, cardio-metabolic disorder, and a higher risk of mental health issues [28]. These earlier results confirm that EMD are a professional group that is at high risk of developing mental health issues and may require special monitoring and surveillance.

Negative correlations were found between FMI scores and HAD Anxiety - Depression, and PCL-5 scores. A positive correlation was found between DM and daytime awareness. Prior research has established an overall positive relationship between DM and psychological well-being [29]. The latter finding suggests that it could be an indicator of psychological health and may be a critical contributor to overall psychological health. Reinforcing DM through mindfulness interventions could be a particularly relevant way for EMD personnel to reduce work-related stress and improve their well-being [30]. Interventions with promising results have been developed for EMD such as a Web-Based Mindfulness Stress Management Program in a corporate call center in Ohio that showed a reduction in perceived stress and increased emotional and psychological well-being for the intervention group compared with a control group.

### Limitations

Our study has several limitations. First, it only explores the prevalence of certain aspects of the occupational health of the call center’s EMD. We did not collect data on burnout or musculoskeletal disorders, which are two pathologies frequently experienced by EMD. Second, we did not conduct a longitudinal study. As data were collected only once, we have no idea if the identified mental health and sleep disorders were present prior to their assignment to the call center, or if they worsened over time. Furthermore, it would probably be better to evaluate PTSD risk as a function of the content of the call. Emergencies are very heterogeneous, and what is experienced as traumatic can vary from one EMD to another, as traumatic situations previously encountered during emergency rescue operations can be reactivated. Developing a better understanding of professional stressors that trigger fear-based reactions to life-threatening situations is a topic for future interventional studies. Finally, our cohort may not be very representative of the general population of EMD, because our sample was almost all male, and former firefighters. These considerations might have increased the disorder cut-off.

In the next step of our work, we will evaluate the impact of a program based on mindfulness on mental health disorders and sleep disorders in EMD personnel working for the PFB.

## Conclusion

The EMD population is at higher risk of developing mental health disorders such as depression and PTSD. Their unique working environment also puts them at risk of physical health problems, and sleep disorders. Mindfulness disposition seems to be a promising resource to improve mental health among EMD. This group of professionals could benefit from specific, multi-level interventions that are tailored to their operational demands, their working environment and their exposure to trauma, and target well-being, mindfulness, and sleep disorders.

## Data Availability

The datasets used and/or analyzed during the current study are available from the corresponding author on reasonable request.
